# The course of health-related quality of life after the diagnosis of childhood cancer: a national cohort study

**DOI:** 10.1186/s12885-023-11379-z

**Published:** 2023-09-11

**Authors:** Marloes van Gorp, Elin Irestorm, Jos W. R. Twisk, Natasja Dors, Annelies Mavinkurve-Groothuis, Antoinette Y. N. Schouten van Meeteren, Judith de Bont, Esther M. M. van den Bergh, Wietske van de Peppel van der Meer, Laura R. Beek, Femke K. Aarsen, Nienke Streefkerk, Raphaele R. L. van Litsenburg, Martha A. Grootenhuis

**Affiliations:** 1 Princess Máxima Center for pediatric Oncology, Utrecht, The Netherlands; 2 Faculty of Medicine, Department of Paediatrics, Lund University, Lund, Sweden; 3 Department of Epidemiology and Data Science, Amsterdam UMC, Location VU University Medical Center, Amsterdam Public Health Research Institute, Amsterdam, The Netherlands

**Keywords:** Childhood cancer, Health-related quality of life, Physical functioning, Emotional functioning, Social functioning, Longitudinal study, Parental distress, Central nervous system tumor

## Abstract

**Background:**

Comprehensive insight in the longitudinal development of health-related quality of life (HRQOL) after childhood cancer diagnosis could improve quality of care. Thus, we aimed to study the course and biopsychosocial determinants of HRQOL in a unique national cohort of children with cancer.

**Methods:**

HRQOL of 2154 children with cancer was longitudinally reported (median: 3 reports) between diagnosis and 5 years after, using the pediatric quality of life inventory generic core scales (PedsQL). HRQOL was modelled over time since diagnosis using mixed model analysis for children 2–7 years (caregiver-reports) and ≥ 8 years (self-reports). Differences in the course between hematological, solid and central nervous system malignancies were studied. Additional associations of demographics, disease characteristics (age at diagnosis, relapse, diagnosis after the national centralization of childhood cancer care and treatment components) and caregiver distress (Distress thermometer) were studied.

**Results:**

Overall, HRQOL improved with time since diagnosis, mostly in the first years. The course of HRQOL differed between diagnostic groups. In children aged 2–7 years, children with a solid tumor had most favorable HRQOL. In children aged ≥ 8 years, those with a hematological malignancy had lower HRQOL around diagnosis, but stronger improvement over time than the other diagnostic groups. In both age-groups, the course of HRQOL of children with a CNS tumor showed little or no improvement. Small to moderate associations (β: 0.18 to 0.67, p < 0.05) with disease characteristics were found. Centralized care related to better HRQOL (β: 0.25 to 0.44, p < 0.05). Caregiver distress was most consistently associated with worse HRQOL (β: − 0.13 to − 0.48, p < 0.01).

**Conclusions:**

The HRQOL course presented can aid in identifying children who have not fully recovered their HRQOL following cancer diagnosis, enabling early recognition of the issue. Future research should focus on ways to support children, especially those with a CNS tumor, for example by decreasing distress in their caregivers.

**Supplementary Information:**

The online version contains supplementary material available at 10.1186/s12885-023-11379-z.

## Background

The overall 5-year survival rates of childhood cancer have increased up to 81% in the Netherlands [1]. Nevertheless, the illness and treatment continue to affect children’s experienced physical, psychological and social functioning, also referred to as health-related quality of life (HRQOL). HRQOL describes the self-perceived impact of illness on a person’s life and is best captured using patient-reported outcome measures (PROMs, also: questionnaires) [2]. Literature reviews show reduced HRQOL in children with cancer from the time of diagnosis to the first years after treatment compared to normative populations [3, 4]. Therefore, a central aim of childhood cancer treatment is optimizing HRQOL throughout and after treatment. To identify individuals who may benefit from intervention, monitoring of HRQOL (using PROMs) is recommended as a standard of care in pediatric oncology [5]. Moreover, the implementation of PROMs in clinical practice can improve patient-physician communication and HRQOL [6].

Comprehensive insight in the course of HRQOL after childhood cancer diagnosis is lacking. Available longitudinal results are from studies with a relatively limited timeframe and/or sample. In children with acute lymphoblastic leukemia (ALL), HRQOL was found to be considerably impaired at 1 month after diagnosis and then improved rapidly, although around 25% still had impaired physical and social functioning 3 months after treatment [7]. In children with non-central nervous system (CNS) cancer, physical HRQOL improved over time from 2 months to 4 years after treatment [8]. In children who received stem cell therapy (SCT), physical and psychosocial HRQOL had improved 1 and 2 years after SCT compared to before SCT [9]. Overall, the results of these studies generally suggest low HRQOL around diagnosis [7], which improves with time [7–9].

In addition to time since diagnosis, other factors were previously found to be associated with HRQOL in children with cancer. Previous literature showed associations between diagnostic groups and HRQOL; children with a CNS tumor were consistently found to have poorer HRQOL [10], and children with leukemia had lower HRQOL than children with a solid tumor [3]. Relapse was found to be associated with worse HRQOL, in particular in children with a CNS or bone tumor [10]. HRQOL was found to be lower while undergoing treatment compared to after treatment completion [10], and specific treatment modalities were also found to be predictive of HRQOL in specific diagnostic subgroups. Cranial radiotherapy was associated with worse HRQOL in children with ALL and children with a high-grade brain tumor [10], and stem cell transplantation was also associated with worse HRQOL in children with ALL [3]. As described in the biopsychosocial model, psychosocial factors impact HRQOL in addition to disease and treatment characteristics [11]. Parent mental health, was found to be an important determinant of HRQOL; symptoms of anxiety, depression and psychological distress were related to poorer child HRQOL [10]. However, family characteristics have been studied to a much lesser extent than diagnosis and treatment factors [10].

In the Netherlands, childhood cancer care includes regular monitoring of HRQOL, resulting in a uniquely large longitudinal data-set of the Dutch cohort of children with cancer. Using this, we aim to (1) describe the course of HRQOL over time in children after childhood cancer diagnosis, (2) determine whether this course differs between diagnostic groups and (3) study the association of biopsychosocial determinants and caregiver distress with HRQOL.

**Table 1:** Descriptives at the first HRQOL assessment

	**Children 2–7 years (n = 1034)**		**Children** ≥ **8 years (n = 1280)**	
	**% or mean**	**n or SD**	**% or mean**	**n or SD**
**Sociodemographic characteristics**				
Proxy gender (male)	24%	247	na	
Parental education level^a^ (Missing: n = 220)				
Low	3%	31	3%	40
Medium	26%	270	21%	270
High	50%	513	38%	481
Missing	21%	220	38%	489
Child sex (male)	56%	574	54%	689
Child current age (years)	4.1	(1.7)	13.3	(3.7)
Range	2.0–8.0		8.0-22.3	
**Diagnosis characteristics**				
Age at first diagnosis (years)	3.2	(1.9)	11.5	(4.2)
< 2	27%	275	4%	52
2–4	39%	407	45%	580
4–7	34%	352	51%	648
Diagnosis after centralization of care^b^	73%	754	63%	807
Time since most recent diagnosis (years), median (IQR)	1.1	(0.5; 2.1)	0.9	(0.4; 2.5)
Diagnosis (*Missing n = 3*)				
*Hematological malignancy*	*45%*	*469*	*48%*	*613*
Acute lymphoblastic leukemia	31%	321	21%	271
Myeloid leukemia	5%	53	6%	70
Langerhans Cell Histiocytosis	5%	47	2%	19
Hodgkin lymphoma	1%	7	12%	156
Non-hodgkin lymphoma	4%	40	7%	95
Other	< 1%	1	< 1%	2
*Solid tumor*	*34%*	*356*	*25%*	*323*
Kidney tumor	12%	124	3%	44
Neuroblastoma	11%	110	2%	23
Soft-tissue tumor	6%	57	7%	83
Germ cell tumor non-CNS	3%	26	3%	39
Bone tumor	2%	15	7%	93
Other	2%	24	3%	41
*CNS tumor*	*20%*	*206*	*26%*	*329*
Low grade glioma	11%	113	12%	154
Ependymoma and choroid plexus papilloma	3%	27	2%	19
Medulloblastoma	3%	26	4%	54
Craniopharyngioma	1%	14	2%	26
High grade glioma	1%	13	2%	26
Germ line tumor CNS	< 1%	2	2%	24
Other	1%	11	2%	26
Missing	< 1%	3	2%	15
Relapsed before assessment	10%	98	12%	152
**Treatment received before assessment**				
Chemotherapy	85%	877	78%	998
Radiotherapy	17%	175	22%	286
Allogenic SCT	1%	14	3%	43
Brain surgery	16%	166	22%	283
Immunotherapy	29%	303	23%	296
**Caregiver clinical distress (DT-P ≥ 4)**				
Yes	51%	524	44%	566
No	42%	440	36%	464
Missing	7%	70	20%	250

SD: standard deviation

CNS: central nervous system

SCT: stem cell therapy

DT-P: distress thermometer for parents

na: not applicable

^a^ Low: primary education, lower vocational education, lower and middle secondary education; medium: middle vocational education, higher secondary education, pre-university education; high: higher vocational education, university

^b^ From 1 to 2014 for solid tumor diagnoses except soft tissue and bone tumors, these and other diagnoses from 15 to 2018

## Methods

### Procedure

Since 2018 all childhood cancer care in the Netherlands is centralized at the Princess Máxima Center for pediatric oncology. This center provides multidisciplinary care and has a focus on the development of the child. Apart from medical treatment, care includes systematic psychosocial support from child life, social work and psychology, as well as supportive care and exercise. Also, HRQOL monitoring and discussion are part of standard care. After opening the Máxima, all families were invited for monitoring, regardless of their time since diagnosis. The KLIK PROM portal monitoring system was previously introduced in 2015 in Dutch pediatric oncology following a long history of development and effectiveness studies to implementation [12, 13]. The KLIK PROM portal is and online system (www.hetklikt.nu) that enables routine monitoring and discussion of electronic PROMs. Monitoring includes assessment of HRQOL and caregiver distress, and is offered every three or six months respectively if the child has an outpatient cancer care appointment. During the appointment the health care professional can display the results of the PROMs in an electronic PROfile and discuss them with the family. Because of the use of clinical data, the number and timing of assessments vary between children. We previously found that approximately 70% of offered questionnaires were completed [12, 14]. Informed consent was obtained from all the study participants, from children themselves (for children aged 12 years and older) and from their parents (for children under age of 16 years). Caregivers also provided informed consent for the use of information on their distress.

**Fig. 1:**
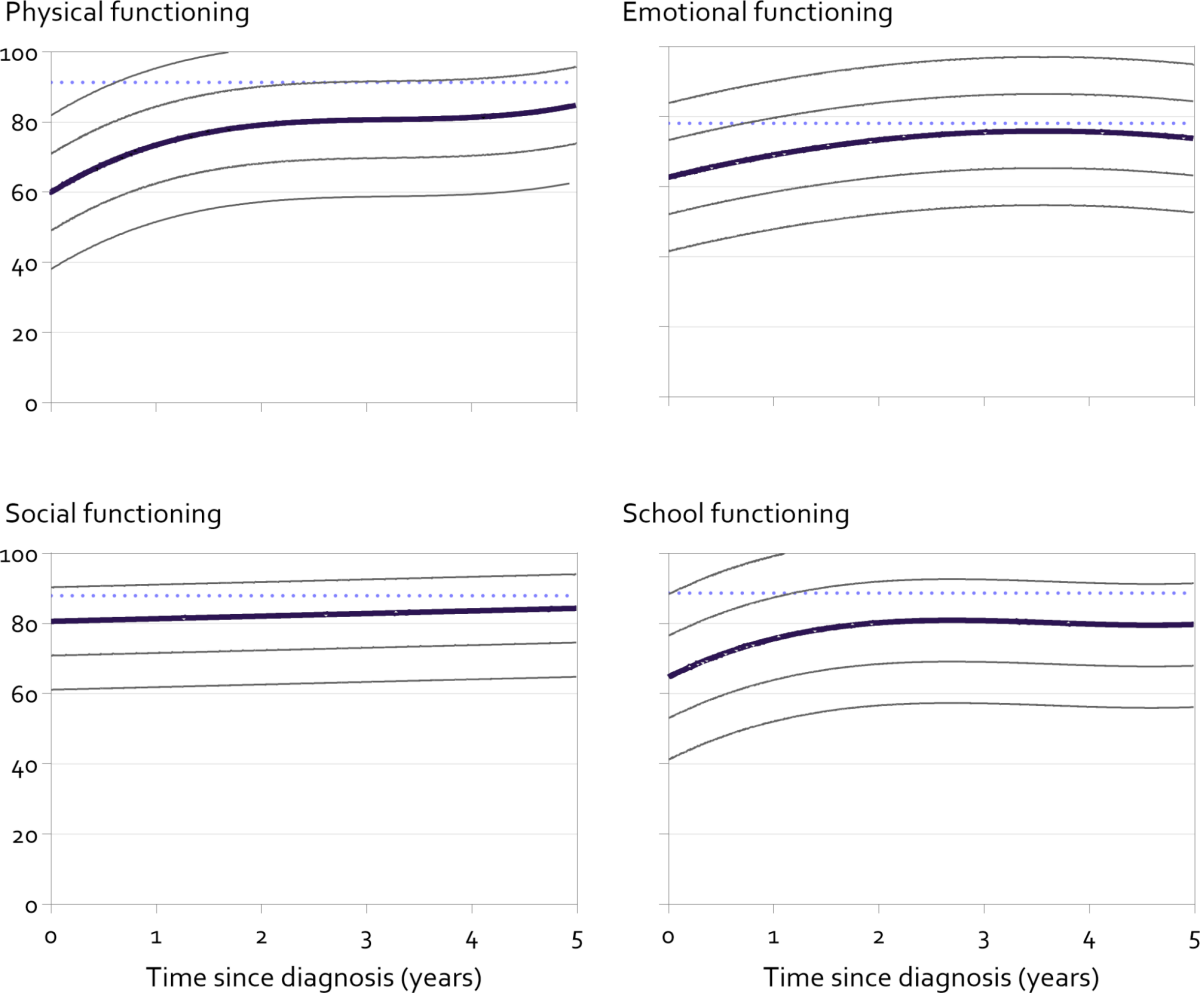
Modelled mean course and 1 and 2 standard deviation lines of physical, emotional, social and school functioning in children 2–7 years old from diagnosis with cancer. SD lines based on residual standard error, calculated as the square root of the sum of squared residual errors divided by the degrees of freedom, i.e.: √(∑(residual)^2^)/df. Dotted line reflects mean general population score, weighted by sex and age of our sample (Schepers 2017)

**Fig. 2:**
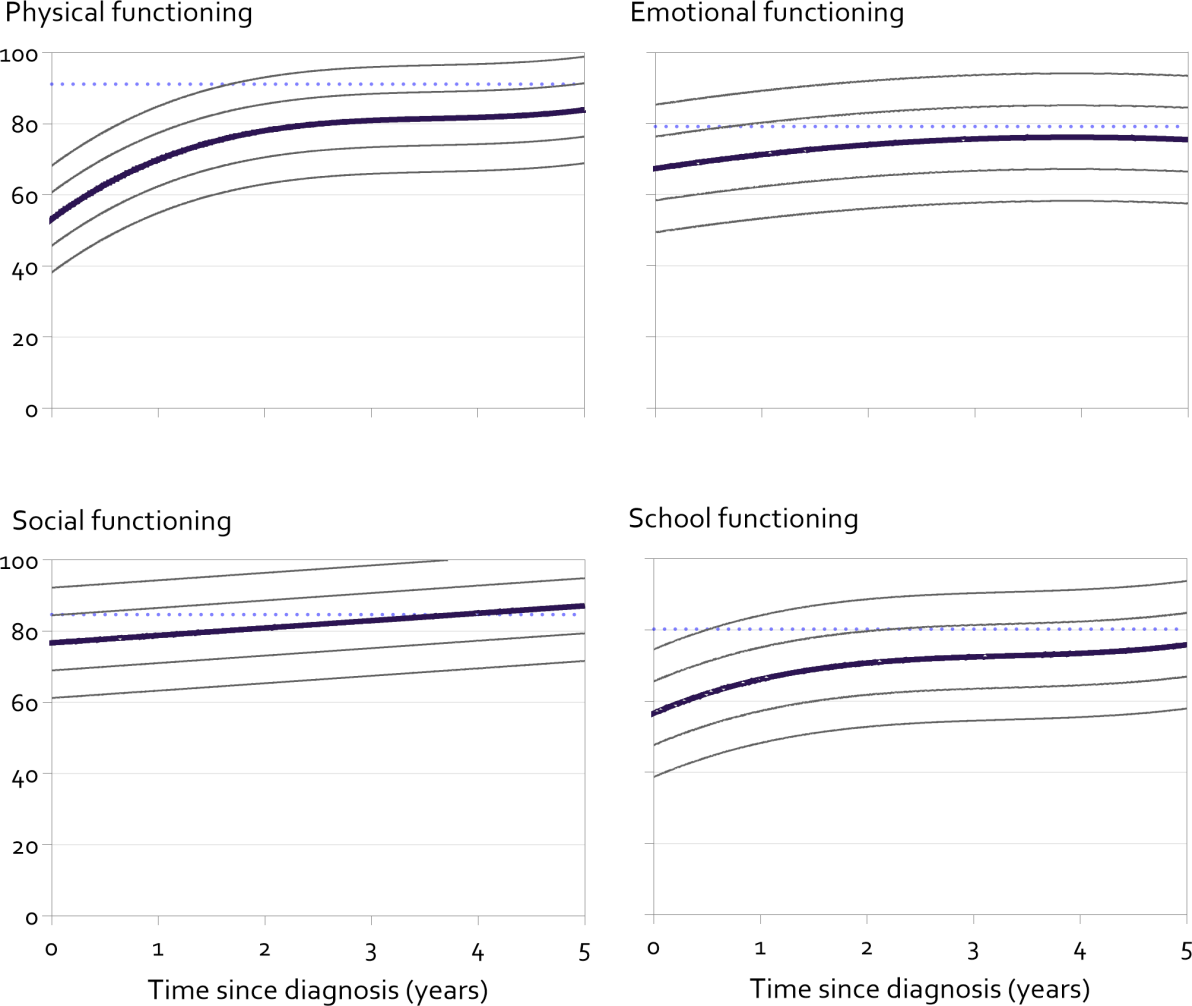
Modelled mean course and 1 and 2 standard deviation lines of physical, emotional, social and school functioning in children  ≥ 8 years old from diagnosis with cancer. SD lines based on residual standard error, calculated as the square root of the sum of squared residual errors divided by the degrees of freedom, i.e.: √(∑(residual)^2^)/df. Dotted line reflects mean general population score, weighted by sex and age of our sample (age 8–18: van Muilekom 2021 and age ≥ 18 Limperg 2014)

**Fig. 3:**
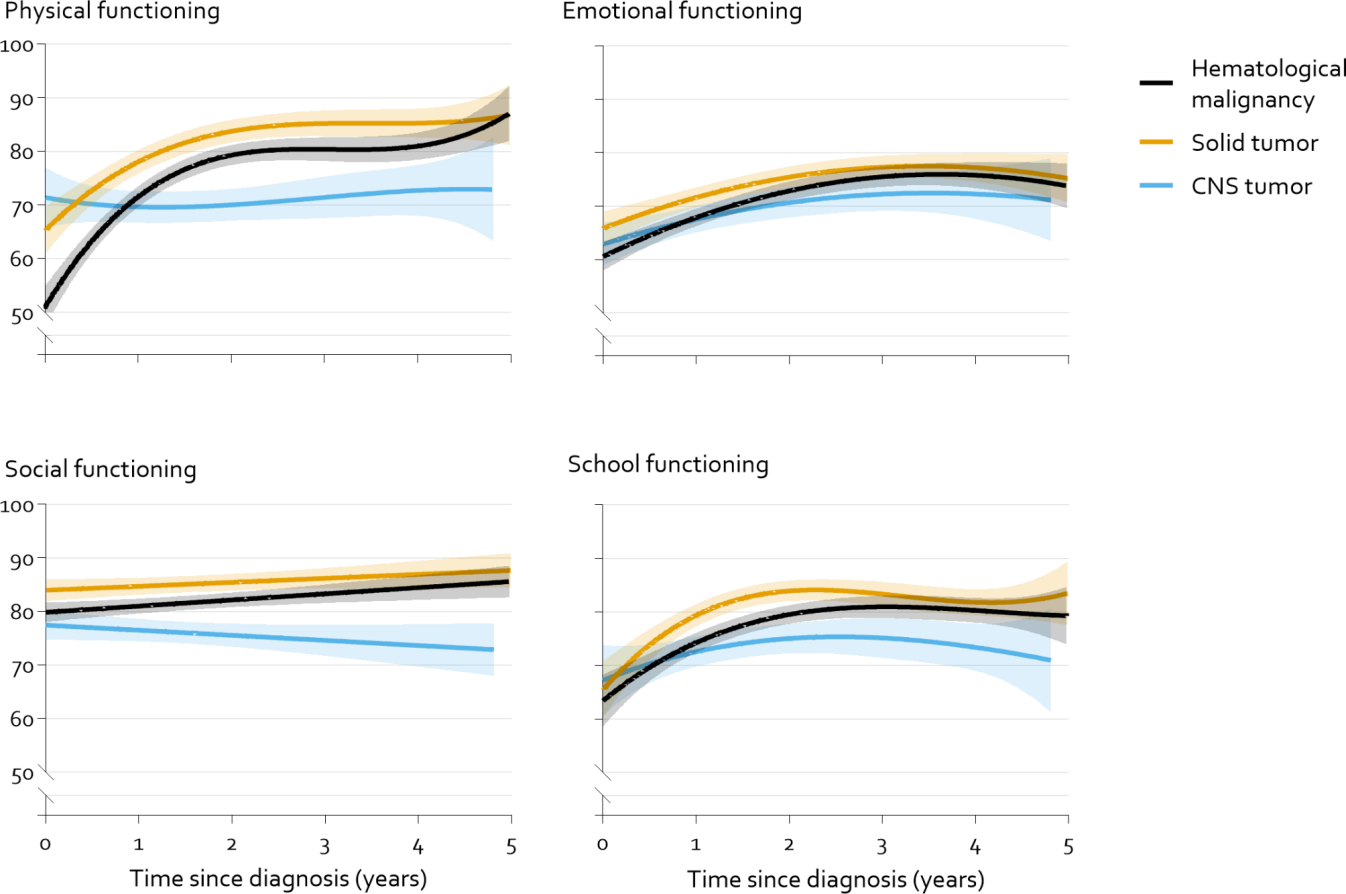
Modelled mean course physical, emotional, social and school functioning of children 2–7 years old by main diagnostic group with 95% Confidence Intervals (shaded). Modelled means are significantly different from each other where the shaded areas do not overlap. The course over time is significantly different between all curves for physical functioning (p < 0.01) and for CNS tumors compared to solid tumors and hematological malignancies in social functioning (p < 0.01). See Supplementary Table 1 for total models

The study was performed in accordance with the declaration of Helsinki. The ethics committee of Rotterdam, The Netherlands (no. MEC-2016-739) approved the use of data collected in care for research questions. Additionally, the scientific committee of the Princess Máxima Centrum approved the project. Inclusion criteria were: (1) receiving care at the Princess Máxima Center for pediatric oncology, or one of the previous pediatric cancer care centers in the Netherlands, (2) taking part in HRQOL monitoring (3) informed consent to use PROM data for research was available. All HRQOL measurements within 5 years since diagnosis that were assessed with the KLIK PROM portal between August 2015 and October 2022 were included.

### Measures

HRQOL of children was assessed using the Dutch pediatric quality of life inventory (PedsQL) core scales. There are separate age-appropriate versions for individuals aged 2–4, 5–7, 8–12, 12–18 and ≥ 18 years with minor differences. Since young children cannot report themselves, caregiver-reported HRQOL was used for children 2–7 years, while children ≥ 8 years reported themselves. The PedsQL assesses HRQOL on four domains: physical functioning, emotional functioning, social functioning and school (or daycare/kindergarten or work/study) functioning. Higher scores (scale: 0-100) indicate higher HRQOL (e.g.: better physical function). Psychometric properties of the Dutch versions of the PedsQL core scales are adequate [15–17]. Cronbach’s alphas of scale scores in this study ranged from 0·79 − 0·90. Dutch general population norm values are available [15, 17, 18].

**Fig. 4:**
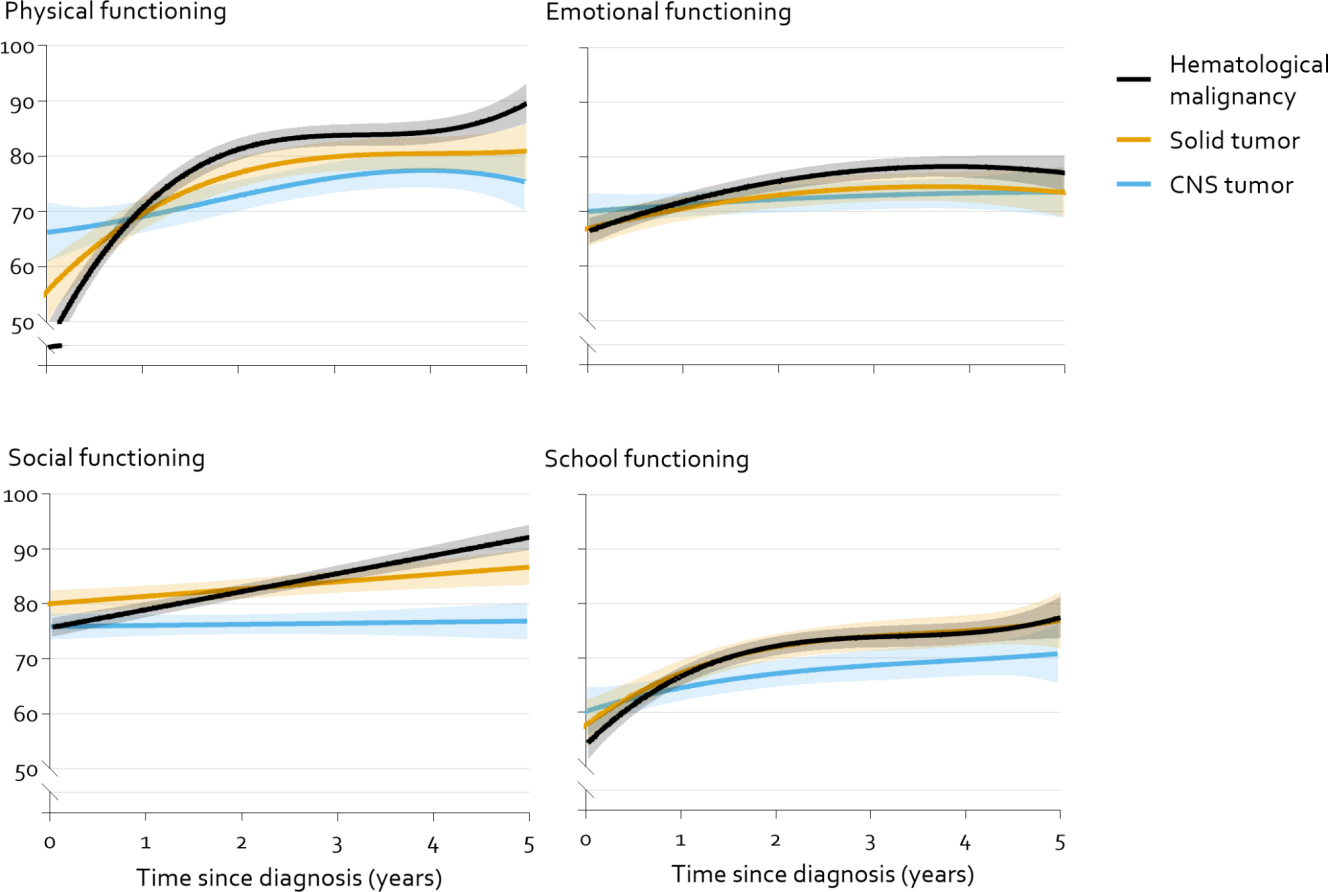
Modelled mean course physical, emotional, social and school functioning of children  ≥ 8 years old by main diagnostic group with 95% Confidence Intervals (shaded). Modelled means are significantly different from each other where the shaded areas do not overlap. The course over time is significantly different between all curves for physical functioning (p < 0.01), between hematological malignancies and CNS tumors for emotional functioning (p = 0.011), between hematological malignancies and the other groups for social functioning (p < 0.001) and between hematological malignancies and CNS tumors in school functioning (p < 0.025). See Supplementary Table 2 for total models

**Table 2:** Standardized Regression coefficients with 95% confidence intervalsf for multivariable determinant models of children 2–7 years old stratified by main diagnostic group

		Physical functioning			Emotional functioning			Social functioning			School functioning		
		HM	ST	CNS	HM	ST	CNS	HM	ST	CNS	HM	ST	CNS
Demographic characteristics ^d^	Current age (4–7 years)		0.02					**− .17** ^**a**^	− 0.01	− 0.06	**− .44** ^**c**^	− 0.08	− 0.19
			(-0.10; 0.15)					(-0.31; − 0.04)	(-0.14; 0.12)	(-0.24; 0.21)	(-0.61; − 0.28)	(-0.23; 07)	(-0.42; 0.03)
Diagnosis characteristics	Age at diagnosis (ref < 2 years)												
	2–4 years	− 0.16	**− .24** ^**b**^		− 0.19	− 0.20		**− .27** ^**b**^	**− .21** ^**a**^	0.25	− 0.14	**− .30** ^**b**^	0.09
		(-0.34; 0.02)	(-0.43; − 0.06)		(-0.39; 0.01)	(-0.39; 0.002)		(-0.47; − 0.07)	(-0.42; − 0.01)	(-0.59; 0.08)	(-0.33; 0.06)	(-0.49; − 0.11)	(-0.20; 0.38)
	4–7 years	− 0.13	**− .27** ^**a**^		− 0.01	− 0.08		**− .25** ^**a**^	**− .27** ^**a**^	**− .67** ^**c**^	− 0.04	**− .33** ^**b**^	**− .39** ^**a**^
		(-0.32; 0.06)	(-0.50; − 0.06)		(-0.22; 0.19)	(-0.30; 0.15)		(-0.48; − 0.02)	(-0.52; − 0.03)	(-1.06; − 0.29)	(-0.27; 0.19)	(-0.57; − 0.09)	(-0.73; .-05)
	Centralized care	**.30** ^**c**^		**.31** ^**a**^				**.25** ^**c**^		**.44** ^**b**^			**.28** ^**a**^
		(0.15; 0.46)		(0.02; 0.60)				(0.10; 0.40)		(0.14; 0.75)			(0.02; 0.54)
	Relapse		**− .26** ^**a**^	− 0.01								**− .37** ^**b**^	
			(-0.46; − 0.06)	(-0.29; 0.26)								(-0.60; − 0.15)	
Treatment characteristics ^d^	Treatment status (on treatment)	− 0.01	**− .22** ^**c**^	− .18^**a**^		− 0.11			− 0.11		− 0.06	− 0.13	− 0.16
		(-0.13; 0.10)	(-0.35; − 0.10)	(-0.33; − 0.02)		(-0.24; 0.02)			(-0.23; 0.001)		(-0.19; 0.06)	(-0.28; 0.02)	(-0.33; 0.02)
	CT	^e^	**− .27** ^**a**^	**− .41** ^**b**^	^e^	− 0.14		^e^	− 0.25	**− .53** ^**c**^	^e^	− 0.05	**− .37** ^**b**^
			(-0.52; − 0.01)	(-0.65; − 0.17)		(-0.43; 0.15)			(-0.53; 0.04)	(-0.77; − 0.28)		(-0.28; 0.24)	(-0.59; − 0.15)
	RT	^e^	− 0.07	**− .52** ^**c**^	^e^			^e^	− 0.02	− 0.14	^e^	− 0.07	− 0.18
			(-0.21; 0.06)	(-0.79; − 0.24)					(-0.17; 0.13)	(-0.42; 0.15)		(-0.23; 0.08)	(-0.43; 0.07)
	Brain surgery	^e^	^e^		^e^	^e^	**− .30** ^**a**^	^e^	^e^		^e^	^e^	
							(-0.58; − 0.02**)**						
	Immunotherapy	**− .21** ^**b**^	− 0.02	− 0.08	− 0.03				− 0.13		− 0.05	− 0.16	
		(-0.36; − 0.05)	(-0.19; 0.16)	(-0.36; 0.20)	(-0.18; 0.11)				(-0.31; 0.06)		(-0.19; 0.09)	(-0.37; 0.05)	
Parental distress	Distress	**− .36** ^**c**^	**− .25** ^**c**^	**− .29** ^**c**^	**− .41** ^**c**^	**− .30** ^**c**^	**− .48** ^**c**^	**− .28** ^**c**^	**− .15** ^**b**^	**− .30** ^**c**^	**− .39** ^**c**^	**− .24** ^**c**^	**− .34** ^**c**^
		(-0.46; − 0.27)	(-0.34; − 0.16)	(-0.45; − 0.14)	(-0.50; 0.32)	(-0.40; − 0.19)	(-0.63; − 0.32)	(-0.37; − 0.18)	(-0.25; − 0.05)	(-0.47; − 0.13)	(-0.49; − 0.28)	(-0.34; − 0.13)	(-0.51; − 0.18)

HM: hematological malignincies, ST: solid tumor, CNS: central nervous system tumor

^a^p<0.05, ^b^p<0.01, ^c^p<0.001

^d^sex and allogenic SCT were not selected for any of the multivariable models

^e^Not studied for this group because of small proportions in the yes or no category (other empty cells indicate the determinants did not have an association with p < 0.1 with the outcome when added to the time model)

^f^Standardized based on SD of all observations. Results in bold have at least a small to moderate effect size (> 0.2) and are significant at p < 0.05 level

^g^Variance of residual error (of model with only correction for individual repeated measures) explained with total model, including the best function of time since diagnosis

HM: hematological malignancies, ST: solid tumor, CNS: central nervous system tumor

^a^p<0.05, ^b^p<0.01, ^c^p<0.001

**Table 3:** Standardized regression coefficients with 95% confidence intervalsf for multivariable determinant models of children ≥ 8 years old stratified by main diagnostic group

		Physical functioning			Emotional functioning			Social functioning			School functioning		
		HM	ST	CNS	HM	ST	CNS	HM	ST	CNS	HM	ST	CNS
Demographic characteristics	Sex (female vs. male)	− 0.09		**− .27** ^a^	− .15^a^	**− .32** ^b^	**− .24** ^a^			**− .34** ^b^			
		(-0.21; 0.04)		(-0.47; − 0.06)	(-0.29; − 0.0003)	(-0.53; − 0.11)	(-0.46; − 0.02)			(-0.57; − 0.11)			
	Current age (ref 8–12)												
	12–18	0.09	− 0.08		.17^b^			0.14	− .20^a^			**− .35** ^b^	− 0.14
		(-0.04; 0.22)	(-0.25; 0.09)		(0.04; 0.29)			(-0.01; 0.29)	(-0.39; − 0.01)			(-0.58; − 0.13)	(-0.30; 0.02)
	18+	0.07	− 0.20		**.28** ^b^			0.17	− 0.21			**− .36** ^a^	− 0.18
		(-0.12; 0.26)	(-0.44; 0.03)		(0.09; 0.48)			(-0.05; 0.39)	(-0.46; 0.04)			(-0.66; − 0.06)	(-0.51; 0.14)
Diagnosis characteristics	Age at diagnosis (ref < 4 years)												
	4–12 years	0.32	0.13			0.47	− 0.06	0.25	0.44	0.20	0.35	0.003	
		(-0.04; 0.68)	(-0.28; 0.53)			(-0.06; 1.00)	(-0.57; 0.45)	(-0.16; 0.66)	(-0.06; 0.94)	(-0.37; 0.77)	(-0.06; 0.76)	(-0.45; 0.45)	
	12–18 years	0.28	0.14			0.48	0.05	0.27	**.54** ^a^	0.12	0.19	0.11	
		(-0.12; 0.68)	(-0.32; 0.59)			(-0.05; 1.01)	(-0.51; 0.61)	(-0.18; 0.72)	(0.001; 1.08)	(-0.49; 0.73)	(-0.22; 0.61)	(-0.41; 0.63)	
	Centralized care	**.35** ^c^	0.16	**.40** ^c^	0.07		0.23	**.39** ^**c**^	0.07	**.34** ^a^			**.29** ^b^
		(0.19; 0.50)	(-0.07; 0.39)	(0.17; 0.63)	(-0.11; 0.24)		(-0.04; 0.50)	(0.22; 0.56)	(-0.18; 0.32)	(0.06; 0.62)			(0.07; 0.52)
	Relapse						− 0.12		.	− 0.11			
							(-0.34; 0.09)			(-0.36; 0.15)			
Treatment characteristics ^d^	Treatment status (during treatment)	**− .31** ^**c**^	**− .24** ^**c**^	**− .22** ^**c**^			− 0.09	**− .22** ^**c**^		− 0.04	− .18^c^	**− .25** ^b^	**− .29** ^**c**^
		(-0.39; − 0.23)	(-0.36; − 0.12)	(-0.33; − 0.10)			(-0.22; 0.03)	(-0.31; − 0.12)		(-0.19; 0.10)	(-0.28; − 0.08)	(-0.41; − 0.09)	(-0.43; − 0.15)
	CT	^e^	**− .45** ^c^	**− .40** ^c^	^e^		− 0.11	^e^	**− .25** ^a^	**− .40** ^b^	^e^		− 0.18
			(-0.69; -20)	(-0.60; − 0.21)			(-0.31; 0.10)		(-0.50; − 0.01)	(-0.64; − 0.16)			(-0.39; − 0.03)
	RT		**.24** ^b^	**− .32** ^b^						− 0.14			− 0.06
			(0.08; 0.41)	(-0.51; − 0.12)						(-0.37; 0.09)			(-0.26; 0.14)
	Brain surgery	^e^	^e^	− 0.16	^e^	^e^		^e^	^e^		^e^	^e^	
				(-0.40; 0.07)									
	Allogeneic SCT	− 0.15	^e^	^e^		^e^	^e^		^e^	^e^	− 0.20	^e^	^e^
		(-0.35; 0.06)									(-0.42; 0.03)		
	Immunotherapy	**− .31** ^c^						**− .25** ^**c**^		− 0.05	**− .24** ^c^	− 0.13	
		(-0.43; − 0.18)						(-0.39; − 0.04)		(-0.42; 0.32)	(-0.37; − 0.10)	(-0.39; 0.14)	
Parental distress	Distress	− .14^c^	− .14^b^	**− .24** ^c^	− .18^c^	**− .30** ^c^	− .19^b^	− .13^b^	− 0.09	**− .29** ^c^	**− .22** ^c^	− 0.05	**− .21** ^b^
		(-0.22; − 0.07)	(-0.25; − 0.03)	(-0.35; − 0.13)	(-0.28; − 0.09)	(-0.43; − 0.17)	(-0.31; − 0.08)	(-0.21; − 0.04)	(-0.20; 0.03)	(-0.42; − 0.15)	(-0.31; − 0.13)	(-0.18; 0.09)	(-0.34; − 0.08)

HM: hematological malignancies, ST: solid tumor, CNS: central nervous system tumor

^a^<.05, ^b^<.01, ^c^<.001

^e^Not studied for this group because of small proportions in the yes or no category (other empty cells indicate the determinants did not have an association with p < 0.1 with the outcome when added to the time model)

^f^Standardized based on SD of all observations. Results in bold have at least a small to moderate effect size (> 0.2) and are significant at p < 0.05 level

^g^Variance of residual error (of model with only correction for individual repeated measures) explained with total model, including the best function of time since diagnosis

#### Older children (≥ 8 years old)

Figure 4 shows the course over time by diagnostic groups for older children (≥ 8 years old). Although children with a hematological malignancy had more impaired physical functioning at diagnosis than children with a solid or CNS tumor (solid tumor: B: 10·1 [SE: 3·5], p = 0·004, CNS tumor: B: 20·9 [SE: 3·5], p < 0·001), and worse social functioning than children with a solid tumor (B: 4·4 [SE: 1·5], p = 0·004), children with a hematological malignancy had stronger improvements in HRQOL than one or both of the other diagnostic groups in all domains. HRQOL of children with a CNS tumor on the other hand, showed the least increase of HRQOL in all domains, and even showed no improvement over time in social functioning. Children with a CNS tumor had lower scores than one or both of the other diagnostic groups in all domains from approximately 2 to 4 years after diagnosis.

### Sociodemographic characteristics

Sociodemographic characteristics of parents were collected in the KLIK PROM portal after registration. If data from both parents was available, the highest level of education was used.

### Disease characteristics

Information on cancer diagnosis (time since diagnosis, diagnostic subgroup, age at diagnosis, being diagnosed before or after the centralization of care and relapse) and treatment were obtained from electronic medical records. Dichotomous variables were created indicating whether a child had relapsed, was undergoing active treatment and had received chemotherapy, radiotherapy, allogenic SCT, brain surgery (information on other types of surgery was unavailable) or immunotherapy at any time before the HRQOL assessment.

### Caregiver distress

Caregivers were asked to report their distress (thermometer score range 0–10, ≥ 4 indicates clinical distress) every six months using the distress thermometer for parents (DT-P). Psychometric properties of the DT-P are good [19]. The response closest in time to the HRQOL assessment was used for analysis.

### Statistical analyses

Distributions of sex, diagnostic group and age at diagnosis were compared to Dutch Childhood Cancer Registry data from 2010 to 2020 using one-sample chi-square tests. This registry includes information on all children with cancer in the Netherlands from 2003 onwards (www.skion.nl). Further analyses were done separately for caregiver-reported (2–7 years) and self-reported (≥ 8 years) outcomes. The course of HRQOL over time was modeled using linear mixed model analyses in SPSS v26. Domains of HRQOL were the dependent outcomes. The main independent variable was time since diagnosis, and quadratic and cubic polynomial time variables were included if significant according to the Wald statistic. The models accounted for within-subject dependency of longitudinal observations using a random intercept and random slopes if significant according to the Likelihood ratio test. First, a total model was built for each outcome. The residual standard error was calculated to provide the mean distance of raw observations to the mean curve, informing standard deviation lines around the modeled mean course. In addition, a line indicating the age and sex- weighted mean of cross-sectional norm data was calculated to provide context to the height of the curves [15, 17, 18]. Second, we studied whether the course of HRQOL was different for diagnostic groups (hematological malignancy, solid tumor, CNS tumor) using dummy variables and interactions with the time variable(s).

Third, the association between HRQOL and demographic characteristics (sex and age at assessment), disease characteristics (age at diagnosis, diagnosis period before or after centralization of care, relapse, treatment), and caregiver distress was studied separately for the diagnostic groups. To adjust the associations for time since diagnosis and repeated individual assessments, best-fitting time models were determined for each diagnostic group. Then, determinants were added one by one. Determinants that were associated with p < 0·1 were selected for final models. To aid interpretation of the regression coefficients of these models, dependent variables were standardized. After Cohen, regression coefficients (β’s) of ·2, ·5 and ·8 were then considered small, medium and large [20]. P < 0·05 was considered statistically significant for all analyses.

## Results

### Participants

Questionnaire data were available from 2341 children (total observations: 8012), and informed consent for research purposes was given for data of 2154 children (92%). Children were mostly male (55%) and the cancer diagnostic group was more often a hematological malignancy (45%) than solid tumor (35%) or CNS tumor (20%). Age at diagnosis was below 4 years in 33%, 4–12 years in 37% and ≥ 12 years in 30% of children. Compared to Dutch Childhood Cancer Registry data, sex was distributed equally, but there were small significant differences in distributions of diagnostic group (44% hematological malignancy, 35% solid tumor, 22% CNS tumor) and age at diagnosis (31% <4 years, 36% 4–12 years, 33% ≥12 years). See Table 1 for more detailed characteristics of the samples for children 2–7 years (n = 1034) and ≥ 8 years (n = 1280). There were 160 children who had observations in both age-ranges and are thus included in the characteristics of both samples. Caregivers reported a median of 3 times (IQR: 2; 5) on the HRQOL of their child (2–7 years), and children ≥ 8 years reported a median of 2 times on their HRQOL (IQR: 1; 5).

### Total course of HRQOL over time for young (2–7 years old) and older (≥ 8 years old) children

Different courses of HRQOL were found for the four domains, that were similar for younger and older children (Figs. 1 and 2 and Supplementary Tables 1 and 2). For physical, emotional and school functioning there was a stronger increase of HRQOL in the first years after diagnosis, that leveled off to a more stable level of HRQOL. For social functioning a rather small (1–2 PedsQL points per year) linear increase was observed over the total range of time (children 2–7 years: B: ·74 [SE: 0·27], p = 0·006, children ≥ 8 years B: 2·08 [SE: 0·23], p < 0·001).

### Course of HRQOL by main diagnostic group

#### Young children (2–7 years old)

Figure 3 shows the course over time by diagnostic group for young children (2–7 years old), that showed significant differences in physical and social functioning. For physical functioning, children with a solid tumor had the most favorable course of HRQOL. Children with a hematological malignancy had lower physical functioning around diagnosis than children with a solid tumor (B: -14·2 [SE: 3·0], p < 0·001), but a stronger increase of HRQOL over time in the first years (p = 0·008). In social functioning, children with a CNS tumor had lower scores around diagnosis than children with a solid tumor (B: -6·5 [SE: 1·7], p < 0·001). For both physical and social functioning, children with a CNS tumor had a different course of HRQOL than the other groups (p < 0·001); their course did not show improvement over time. The course over time of emotional functioning and school functioning was not significantly different between diagnostic groups, but children with a solid tumor had a little better emotional functioning around diagnosis than children with a hematological malignancy (B: 5·3 [SE: 2·1], p = 0·011) and better school functioning than children with a hematological malignancy or a CNS tumor from 1 to 2·5 years after diagnosis.

### Biopsychosocial determinants of HRQOL

Tables 2 and 3 show the independent associations between demographic and disease characteristics and caregiver stress with HRQOL in children 2–7 years old (Table 2) and ≥ 8 years old (Table 3). Sex was not significantly associated with HRQOL in young children, but ≥ 8 years old girls had lower HRQOL than boys, especially in children with a CNS tumor. A higher age at assessment was related to lower HRQOL in school functioning in some subgroups, but also to better emotional functioning (≥ 18 years vs. 8–12 years). Age between 2 and 7 years at diagnosis was related to lower HRQOL than < 2 years, mostly in social functioning. Being diagnosed in the teens, on the other hand, was related to higher social functioning compared to being diagnosed at a young age (< 4 years). A diagnosis after the national centralization of care was associated with better HRQOL, mostly in physical and social functioning. Relapse was only associated with lower HRQOL in young children with a solid tumor. Children had lower HRQOL during treatment, but effect sizes were modest, especially in young children. Out of the treatment factors, HRQOL was mostly associated with having had chemotherapy. From the analyses in all three diagnostic groups on the domain emotional functioning, brain surgery in young children with a CNS tumor was the only diagnosis or treatment characteristic that was associated with HRQOL. Caregiver distress was consistently associated with lower HRQOL in young children, and in most diagnostic groups of older children as well although with smaller effect sizes.

## Discussion

In this unique national cohort study, we provided comprehensive insight in the course of HRQOL over a 5-year period for children with cancer. HRQOL increased with time since diagnosis, and for most domains this increase was strongest in the first years. The course of HRQOL differed between diagnostic groups and was least favorable for children with a CNS tumor. Finally, we studied associated factors and found that caregiver distress was consistently associated with HRQOL, while demographic characteristics and diagnosis and treatment characteristics were associated with specific HRQOL domains or in diagnostic groups.

The overall improvement of HRQOL over time since diagnosis that we found is in line with the literature of subgroups of children with cancer [7–9]. The average levels of HRQOL of almost all domains remain below that of general population peers from diagnosis throughout the first five years (see Figs. 1 and 2), except for adolescent social functioning from 3 years after diagnosis [15, 18]. Also, the average level of emotional functioning of children with cancer approaches that of references from around 3 years after diagnosis [15, 18]. Since the assessments were collected as part of the implementation of patient reported outcome measures in clinical practice, this may have positively influenced the levels of HRQOL [21]. Further implementation of PROMs in clinical practice is recommended to optimize HRQOL.

Our results of this large cohort study provide reliable trajectories of HRQOL in the current Dutch pediatric oncology setting. Future studies and trials may use the presented modelled mean course or means at different time points to determine whether their results differ from the Dutch current standard of care. Healthcare professionals may use the mean curves with standard deviations to see whether an individual patient is outside of 68% or 95%-ranges of their diagnostic peers at different times since diagnosis. For example, these pediatric oncology-specific reference values may be shown when discussing HRQOL in consultations. This may augment existing benefits of using patient reported outcome measures in clinical practice, like improved communication, satisfaction with care and improved HRQOL [21–24].

The results emphasize that children with a CNS tumor are a risk group, as their HRQOL does not seem to improve over time like that of other diagnostic groups. Lower HRQOL in the diagnostic group of children with a CNS tumor was previously found in cross-sectional studies [10]. Our results on a stable course of physical and social functioning suggest that attention is warranted for children with a CNS tumor with low HRQOL early on, as this may not spontaneously improve over the course of the first years. Intervention could for example consist of social skills training and/or a (group-based) active play program offered during treatment, which targets both domains of physical and social functioning [25, 26]. In addition to intervention, adapting a child’s environment (such as: reducing amount or level of school tasks, or offering adaptive devices) may improve their (preschool or school) participation and HRQOL.

The associations between sex and age with HRQOL are in line with those found in the general population. In young children, sex is not related to HRQOL, while in older children girls have poorer HRQOL than boys [15, 18]. The lower scores in school functioning of older children compared to younger children are also in line with the general population, and likely reflect differences in questionnaire versions and increasing demands with age in this domain (i.e. going to school or having work) [15].

The reports of HRQOL were higher after the centralization of pediatric oncology care in the Netherlands. The program of the exercise and psychosocial team may have contributed to these results, but also the generally higher concentration of expertise, a focus on developmentally oriented care, or improving HRQOL as part of the center’s core mission. Also the multidisciplinary collaboration including rehabilitation medicine or having a dedicated pain team may have improved the domain physical functioning (which includes an item on pain).

Several treatment modalities showed some negative associations with HRQOL. Most consistently we found that having had chemotherapy impacted HRQOL. A large majority of children with cancer receives chemotherapy, and the absence of chemotherapy therefore may reflect having received a less intensive treatment, which may explain their better HRQOL. Similarly, the negative effect of immunotherapy on HRQOL that was found in children with a hematological malignancy may be explained by immunotherapy currently being offered more often to children with higher risk disease. Future research may focus on different types of immunotherapy and their independent effect on HRQOL. We only found an association of brain surgery to emotional functioning in young children, but it should be taken into account that this variable included any surgical procedure on the brain (e.g. including diagnostic biopsies), making it less discriminative to the impact of surgery.

We found that caregiver distress was systematically associated with their child’s HRQOL. It is important to be aware of the caregiver-reporting bias that may partially explain this in the younger children (i.e. distressed caregivers report lower HRQOL for their children) and that effect sizes were modest in the older child cohort. Nevertheless, it stresses the relevance of taking the caregiver’s functioning into account in pediatric oncology, and may signal the importance of the family in general. Interventions to prevent or reduce distress of the caregivers, such as psychoeducation or cognitive behavioral based interventions aimed at teaching coping strategies [27], may potentially improve children’s HRQOL as well. The psychosocial assessment tool may be used to guide the intensity of the necessary interventions for a family [28].

In emotional functioning, the course of HRQOL showed the most overlap between diagnostic groups and few associations were found between medical characteristics and emotional functioning. This suggests that the impact of cancer diagnosis and treatment trajectory on emotions such as sadness or worry is rather generic. This is in line with the pediatric medical stress model which describes that events that cause pediatric medical stress are mostly unrelated to disease group [29].

### Limitations

It is important to acknowledge that non-Dutch speaking families were not approached for monitoring. We are currently improving inclusiveness of the monitoring by implementing English questionnaire versions. Representativeness of the cohort seems to be further limited by an underrepresentation of caregivers with lower or medium level of education compared to the Dutch general population. The impact of this response bias on the results is uncertain, as the literature both describes that parental education is unrelated to child HRQOL, and that higher parental education relates to better child HRQOL [10]. Since our study had too many missing cases on the parental education variable, we were unable to analyze the impact properly in our study. Furthermore, when children turn 8, they self-report their HRQOL for care purposes, and caregivers no longer fill out proxy-report questionnaires. Because of this, the course of HRQOL for children 2–7 years is less representative for the total cohort at the end of the timeframe, as this only includes data of children who were relatively young at diagnosis. Finally, future research may add insight to the association of other psychosocial factors such as coping styles and social support with the course of HRQOL in different domains.

## Conclusions

In children with cancer, HRQOL increases in all domains from time since diagnosis. For children with a CNS tumor, however, the course over time showed little or no improvement. The presented courses of HRQOL over time can be used for early recognition of patients at risk for insufficient recovery of HRQOL. Future research should focus on ways to improve HRQOL in those children, especially those with a CNS tumor. Centralized multidisciplinary care seems to have positive effects on the HRQOL of children. HRQOL intervention may be aimed at optimizing specific supportive care in the first years after diagnosis, but also at reducing caregiver’s stress.

### Electronic supplementary material

Below is the link to the electronic supplementary material.

Supplementary Material 1

## Data Availability

The datasets used and/or analysed during the current study available from the corresponding author on reasonable request.

## List of Abbreviations

HRQOL: Health-related quality of life
PROM: Patient-reported outcome measure
CNS: Central nervous system
ALL: Acute lymphoblastic leukemia
PedsQL: Pediatric quality of life inventory generic core scales
DT-P: Distress thermometer for parents
