# Supplementary material for: The course of health-related quality of life after the diagnosis of childhood cancer: a national cohort study
Source: BMC Cancer. 2023 Sep 11;23:855. doi: 10.1186/s12885-023-11379-z (PMC10496372; doi:10.1186/s12885-023-11379-z)
Supplement: Supplementary file 1 — Supplementary Material 1 [file 12885_2023_11379_MOESM1_ESM.docx]

| **Supplementary table 1: Regression coefficients of course of physical, emotional, social and school functioning of children 2-7 years old (Figure 1 and 3)** | | | | | | | | |
| --- | --- | --- | --- | --- | --- | --- | --- | --- |
|  | Physical functioning | | Emotional functioning | | Social functioning | | School functioning | |
|  | Total course | Main diagnostic groups | Total course | Main diagnostic groups | Total course | Main diagnostic groups | Total course | Main diagnostic groups |
| Intercept | 60.02^c^ (1.34) | 50.97^c^ (1.95) | 62.74^c^ (0.92) | 60.53^c^ (1.36) | 80.65^c^ (0.62) | 79.86^c^ (0.91) | 64.77^c^ (1.61) | 63.35^c^ (2.49) |
| Time since diagnosis (years) | 18.56^c^ (2.01) | 29.04  (2.92)^c^ | 7.37^c^ (0.85) | 8.50^c^ (1.25) | 0.74^b^ (0.27) | 1.15^b^ (0.39) | 14.92^c^ (2.34) | 14.24^c^ (3.59) |
| Time^2^ | -5.65^c^ (0.95) | -9.50 (1.39)^c^ | -1.03^c^ (0.18) | -1.17^c^ (0.26) |  |  | -4.39^c^ (1.07) | -3.67^b^ (1.62) |
| Time^3^ | 0.59^c^ (0.13) | 1.03  (0.19)^c^ |  |  |  |  | 0.40^b^ (0.15) | 0.29 (0.22) |
| Hemato (ref) |  |  |  |  |  |  |  |  |
| Solid tumor |  | 14.24^c^ (2.99) |  | 5.30^a^ (2.09) |  | 4.11^b^ (1.38) |  | 2.01 (3.68) |
| CNS tumor |  | 20.44^c^ (3.49) |  | 2.33 (2.47) |  | -2.40 (1.67) |  | 3.83 (4.19) |
| Time*Hemato (ref) |  |  |  |  |  |  |  |  |
| Time*Solid |  | -11.71^c^ (4.37) |  | -1.75 (1.88) |  | -0.41 (0.58) |  | 5.82 (5.24) |
| Time*CNS tumor |  | -32.27^c^ (5.54) |  | -3.07 (2.44) |  | -2.10^b^ (0.78) |  | -7.26 (6.43) |
| Time^2^*Hemato (ref) |  |  |  |  |  |  |  |  |
| Time^2^*Solid |  | -4.51^a^ (2.05) |  | 0.20 (0.39) |  |  |  | -3.13 (2.36) |
| Time^2^*CNS tumor |  | -11.14^c^ (2.80) |  | 0.40 (0.55) |  |  |  | 1.96 (3.13) |
| Time^3^*Hemato (ref) |  |  |  |  |  |  |  |  |
| Time^3^*solid tumor |  | -0.55 (0.28) |  |  |  |  |  | 0.41 (0.32) |
| Time^3^*CNS tumor |  | -1.22^b^ (0.41) |  |  |  |  |  | -0.20 (0.45) |
| ^a^p<.05, ^b^p<.01, ^c^p<.001 | | | | | | | | |

**Supplementary material**

Table of contents

p. 1: Supplemental table 1: Regression coefficients of course of physical, emotional, social and school functioning of children 2-7

years old (Figure 1 and 3)

p. 2: Supplemental table 2: Regression coefficients of course of physical, emotional, social and school functioning of children ≥8 years old (Figure 2 and 4)

| **Supplementary table 2: Regression coefficients of course of physical, emotional, social and school functioning of children** ≥**8 years old (Figure 2 and 4)** | | | | | | | | |
| --- | --- | --- | --- | --- | --- | --- | --- | --- |
|  | Physical functioning | | Emotional functioning | | Social functioning | | School functioning | |
|  | Total course | Main diagnostic groups | Total course | Main diagnostic groups | Total course | Main diagnostic groups | Total course | Main diagnostic groups |
| Intercept | 53.25^c^  (1.45) | 45.33^c^ (2.01) | 67.38^c^ (0.86) | 66.35^c^ (1.22) | 76.76^c^  (0.62) | 75.64^c^ (0.89) | 56.64^c^ (1.14) | 54.07^c^ (1.61) |
| Time since diagnosis (years) | 22.09^c^ (2.10) | 34.54^c^ (2.87) | 4.50^c^ (0.77) | 6.24^c^ (1.08) | 2.08^c^ (0.23) | 3.29^c^ (0.31) | 12.80^c^ (1.72) | 17.04^c^ (2.42) |
| Time^2^ | -5.92^c^  (0.92) | -10.40^c^ (1.26) | -0.57^c^ (0.16) | -0.82^c^  (0.22) |  |  | -3.54^c^ (0.80) | -5.00^c^ (1.13) |
| Time^3^ | 0.55^c^  (0.12) | 1.05^c^  (0.16) |  |  |  |  | 0.35^b^ (0.11) | 0.51^b^  (0.15) |
| Hemato (ref) |  |  |  |  |  |  |  |  |
| Solid tumor |  | 10.05^b^  (3.47) |  | 0.55 (2.09) |  | 4.40^c^  (1.51) |  | 3.67 (2.78) |
| CNS tumor |  | 20.86^c^  (3.47) |  | 3.66 (2.11) |  | 0.22  (1.52) |  | 6.13^a^  (2.78) |
| Time*Hemato (ref) |  |  |  |  |  |  |  |  |
| Time*Solid |  | -16.32^b^ (4.99) |  | -2.03  (1.86) |  | -1.96^c^ (0.54) |  | -4.62 (4.16) |
| Time*CNS tumor |  | -32.64^c^ (5.04) |  | -4.86^a^  (1.92) |  | -3.09^c^  (0.55) |  | -11.64^b^ (4.26) |
| Time^2^*Hemato (ref) |  |  |  |  |  |  |  |  |
| Time^2^*Solid |  | 5.97^b^ (2.20) |  | 0.24 (0.37) |  |  |  | 1.74 (1.94) |
| Time^2^*CNS tumor |  | 11.60^c^ (2.23) |  | 0.68 (0.39) |  |  |  | 3.82 (2.00) |
| Time^3^*solid tumor |  | -0.69^a^  (0.28) |  |  |  |  |  | -0.20  (0.26) |
| Time^3^*CNS tumor |  | -1.29^c^  (0.29) |  |  |  |  |  | -0.40  (0.27) |
| ^a^p<.05, ^h^p<.01, ^c^p<.001 | | | | | | | | |
